# Quality of life of women who practice dance: a systematic review protocol

**DOI:** 10.1186/s13643-018-0750-5

**Published:** 2018-07-10

**Authors:** Janete Capel Hernandes, Viviane Cruvinel Di Castro, Mauro Elias Mendonça, Celmo Celeno Porto

**Affiliations:** Faculdade de Medicina. Programa Ciências da Saúde, Universidade Federal de Goiás, Secretaria–1a s/n–Setor Universitário, Goiânia, Goiás 74605-020 Brazil

**Keywords:** Quality of life, Women, Dance, Systematic review, Protocol

## Abstract

**Background:**

As general population life expectancy has increased, the need to investigate the quality of life has arisen, especially because it is important that people have a healthy long life and with good quality. Studies are done with specific populations, and in the case of this investigation, the target studies are the ones done with women. Female population is growing demographically and professionally. Women have shown increased levels of stress and higher number of illness. It is known that many practices can be used to improve the level of quality of life and that one of them is the dance. Dance is an activity which combines physical and psychosocial aspects. Moreover, it promotes self-expression, self-esteem, and self-confidence. It relieves women of stress. And it also helps in a variety of aspects such as group interaction, motivation, and positive emotions. In this systematic review, the main objective is to assess the effect of dance on quality of life of adult women.

**Methods:**

Only quantitative studies will be included. Studies will be accepted with any amount of dance practice time. They will have been published in the following bibliographic databases: Scientific Electronic Library Online (SciELO), Biblioteca Virtual em Saúde (BVS), Portal da Coordenação de Aperfeiçoamento de Pessoal de Nível Superior (CAPES), MEDLINE, Embase, and Cochrane from inception until June 30, 2018. There will be no restrictions for geographical location. It will be included studies published in Portuguese, English, and Spanish. Only published ones will be included in the review.

**Discussion:**

There is a variety of systematic review studies with men and women on quality of life and dance, but this is the first one focused exclusively on adult female audience. It is expected that this review will be useful to promote the discussion about quality of life of adult women and the interest of this population for dance practice. In order to summarize and to explain the characteristics and findings of those studies, tables and information from texts will be used in a systematic narrative synthesis.

**Systematic review registration:**

PROSPERO CRD42016039961

**Electronic supplementary material:**

The online version of this article (10.1186/s13643-018-0750-5) contains supplementary material, which is available to authorized users.

## Background

Women’s professional growth and their increasing numbers are sensed in several countries around the world. This growth has been followed by changes in the construction of identity, social roles, and health [1, 2].

World Health Organization (WHO) reveals that in 2013, the main death causes in adult women were HIV/AIDS (12.3%), heart disease (6.9%), brain vascular accident—stroke (6.6%), tuberculosis (5%), breast cancer (4%), suicide (3%), cervical cancer (2.7%), respiratory infections (2.6%), traffic accidents (2.5%), and lung disease (2.4%) [3].

According to the Federação Brasileira das Associações de Ginecologia e Obstetrícia (FEBRASGO), in 2011, more than 43 thousand Brazilian women died from cardiovascular disease, 41.9% of cases, whereas 50 years ago, the rate was 10%. This institution ascribes this increase to the fact that women were forced to enter in the labor market and to fight for equality in it. They accumulated several functions (they work inside and outside their houses). In many occasions, they are responsible for the house expenses. As a result, the level of stress has been increasing considerably and affecting directly their quality of life [4].

Even though quality of life has been a widely researched topic, it is still relevant considering the increase of life expectancy. Because of that, the need to investigate quality of a longer life has risen. According to medicine advances, the concept of life quality was emerged in the 1970s. Moreover, they allowed an extension of life and a possibility of an evaluation or measurement of it. It is understood that life quality describes not only a person’s health condition but also aspects related to the general environment. It is a subjective concept which involves the perception of pleasant and unpleasant sensations felt by an individual in relation to his/her reality [5].

To World Health Organization (WHO), quality of life can be regarded as the way an individual realizes his/her position in life and culture. Therefore he/she becomes aware of his/her satisfaction, expectations, living standards, and concerns [6].

In order to optimize people’s quality of living, there is a need to find effective, enjoyable, and sustainable methods of exercising. Dancing is a popular exercise, mainly among women [7]. Dance can be considered as an activity that combines physical and psychosocial aspects. It promotes self-expression, self-esteem, self-confidence, stress relief, group interaction, motivation, and positive emotions [7–9].

Dance has been around since ancient times, and its history is intertwined with mankind’s history. It was created to fulfill the need to revere the divine, the sacred. Most dances were originated in ritualistic contexts in which people worshipped nature-related gods. Dance can be perceived as an art of moving the body through time and space, caused by the choreographic rhythms [10].

Dance can provide a great sense of pleasure and lead the individual to find harmony and a sense of belonging. In addition to that, it influences in a positive way on the individual’s socialization. When the internal desire to move is satisfied, it promotes and improves health not only physically, but also mentally, emotionally, and socially [11].

Studies have been carried out in many parts of the world on quality of life and dance [12–14]. They have shown that dance can interfere positively on improving health and well-being and mainly in the quality of life of people with different sociodemographic characteristics.

These studies have also revealed the application of various styles of dance as ancillary techniques in the treatment of patients with diseases such as Parkinson, fibromyalgia, cancer, hypertension, schizophrenia, rheumatoid arthritis, and multiple sclerosis.

There is growing recognition among healthcare providers and researchers that promoting physical exercise at early stages in life and after diagnosis and treatment of diseases is an amazing option because it could prevent death and extend life expectancy. Research needs to focus on quality of life, specially using new methods, new recommendations, and a combination of non-pharmacological and pharmacological treatment modalities [14–17].

In this way, according to most of the studies, the practice of dance could be associated with a better quality of life [14–17]. Although a great amount of studies about the influence of dance on people’s quality of life exists, it is necessary to evaluate the specificity and the quality of these research.

It is known that there are systematic reviews dedicated to the study of the effect of various types of dance in aid of treatment of many diseases [18–20] and also focused on the study of quality of life and dance [21, 22]. However, there are no systematic reviews relating to dance practice and quality of life of adult women.

One systematic review protocol [23] relates quality of life of adult women who engaged in physical exercises, but it does not focus specifically on dance. Therefore, our proposal is to assess the effect of dance on quality of life of adult women.

### Objectives

The objectives of this systematic review are:

1. Verify the influence of dance on quality of life of adult women;
2. Compare life quality among adult women who practice dance to those who do not practice any type of it;
3. Combine the statistical results of the primary studies in a meta-analysis, if appropriate.

## Methods

We will perform a systematic review of studies exploring the quality of life of women practicing dance. The review was recorded in PROSPERO [24] database (registration number CRD42016039961). This protocol was structured according to the Preferred Reporting Items for Systematic Review and Meta-Analysis Protocols (PRISMA-P) [25] guidance (see Additional file 1).

### Eligibility criteria

Studies will be selected according to the criteria outlined below:

#### Participants

Studies which include adult women, according to the criteria described by the World Health Organization (WHO) (20 to 64 years old) [26], with or without any type of health problem. Studies with children, adolescents, and older women will be excluded.

#### Study designs

Only published randomized and quasi-randomized controlled trials and studies in which generic or specific quality of life tools were used are to be included. Qualitative studies will be excluded.

#### Interventions or exposure

The intervention or exposure of interest is the practice of any kind of dance.

#### Comparators

A comparison will be made with women who do not practice any kind of dance.

### Outcomes

#### Primary outcome

The primary outcomes to be considered are the quality of life indicators described in the studies. These indicators can be found in some instruments for assessing quality of life. We highlight two of them: SF-36 (Medical Outcomes Study 36-Item Short-Form Health Survey*)* [27] and World Health Organization Quality of Life Measures (WHOQOL-BREF) [28]. In the first instrument of quality of life, indicators are related to the following dimensions: physical functioning, bodily pain, general health, social and emotional aspects, and mental health. In the second instrument, the indicators are related to four domains, physical, psychological, social affairs, and the environment, and two items related to global quality of life and status of general health. Indicators described in other instruments that assess quality of life will also be considered in the analysis of the data. We will not use studies with no data collection instrument to assess quality of life. It will be not enough to simply refer to the term quality of life.

#### Secondary outcomes

Secondary outcomes will be not considered in this review.

### Search methods

Literature search strategies will be developed using Medical Subject Headings (MeSH), Descritores em Ciências da Saúde (DeCS), Embase Thesaurus (Emtree) [29], and text words. We will search MEDLINE [30], Scientific Electronic Library Online (SciELO) [31], Biblioteca Virtual em Saúde (BVS) [32] database, Cochrane Library catalog [33], Coordenação de Aperfeiçoamento de Pessoal de Nível Superior (CAPES) [34] data, and Embase. The data will be reached from inception until June 30, 2018.

To ensure literature saturation, we will scan the reference lists of included studies or relevant reviews identified through the search. We will also search the authors’ personal files and get in contact with them, if necessary. For searches in the databases, the following keywords or search terms are used: “Quality of life and dance” and “Quality of life” and “women” and “dance”. The data found in the search process requires the use of specific software for this process step. For this, it will be used with an EndNote reference manager. If repeated studies are identified, they will be removed from the research. Only studies with any amount of dance practice time, in Portuguese, English, and Spanish, in any geographical location, will be accepted. An example of the final research strategy used for the MEDLINE database is provided (see Additional file 2).

### Selection of studies

Two reviewers will independently screen the studies identified by the searches following a three-phase procedure. After each phase, the reviewers will check inclusions and exclusions, and, in case of disagreements, a third reviewer will be involved as an adjudicator.

For phase 1, title of articles identified by the searches will be screened against the following criteria:

1. Is the study with adult women? (Yes, not clear, or no)
2. Is the study related to dance? (Yes, not clear, or no)
3. Is it a study of quality of life? (Yes, not clear, or no)

For phase 2, abstracts of the studies selected on phase 1 will be read and screened against the following criteria:

1. Is the study with adult women? (Yes, not clear, or no)
2. Is the study related to dance? (Yes, not clear, or no)
3. Is it a study of quality of life? (Yes, not clear, or no)
4. Is it a randomized or quasi-randomized controlled trial? (Yes, not clear, or no)
5. Is the study in Portuguese, English, or Spanish? (Yes, not clear, or no)

For phase 3, we will read full texts of the studies meeting all the above inclusion criteria and elaborate a table with the following information:

1. Authors’ names
2. Title of the study
3. Journal title
4. Keywords
5. Journal impact factor
6. Date of the study publication
7. Country of origin
8. Study publishing language
9. Summary of studies containing the following information: participants, intervention, comparison, outcomes, study type, study design, instruments used to evaluate participants’ quality of life, domains contained in quality of life instruments, limitations and relevance of the studies, reasons women practice dance, dance styles that are practiced by the participants, their health status, risk of study bias, and method of analysis of the results.

We will get complete reports of all the studies that appear to meet the inclusion criteria, or when there is some uncertainty.

Reviewers then examine the full text reports and decide whether the inclusion criteria are definitely presented. Additional information from the authors of the study will be sought, if necessary, to solve questions about eligibility. Disagreements between reviewers regarding the eligibility of particular studies through a collective discussion will be resolved by including a third reviewer. Reasons for the studies’ exclusion will be recorded. None of the review authors will be blind to the journal titles or authors or study institutions. A flow diagram of the study will be done containing measures, such as the identification, screening, eligibility and inclusion of items with quantities, and an explanatory statement on the grounds of exclusion.

### Data extraction and management

Using standardized forms and a detailed instruction manual, which will be used to inform specific tailoring of an online data abstraction program (EndNote reference manager), reviewers will be able to extract data independently and duplicate from each eligible study. Data abstracted will include demographic information, methodology, intervention details, and outcomes. If data is available, the following criteria will be inquired: studies’ name of authors, journal title, date of elaboration of the article and its publication date; country of origin of studies; the instruments used to evaluate participants’ quality of life; women’s socioeconomic profile in the study; and the reasons women practice dance and styles that are practiced by them. In case of possible differences found in the quality of life tools, they will be analyzed by grouping the same or similar domains of the instruments. Consequently, an analysis of the results of each domain will be done. Reviewers will resolve disagreements by discussing, and one arbitrator will adjudicate unresolved ones. Study authors will be contacted to resolve any uncertainties. A data extraction technique to obtain outcome data not reported in a usable format into a more useful format for the research will be used. In case of missing information, reviewers will try to contact authors of included studies with a maximum of three e-mail attempts. In order to avoid overlapping reports, a method to identify and deal with multiple reports of a single study will be developed.

### Quality assessment

To assess the risk of bias in non-randomized included studies, ROBINS-I tool will be used. The response options for each domain level will be: “low risk,” “moderate risk,” “serious risk,” “critical risk,” and “no information” [35]. To assess the risk of bias of randomized included studies, the Cochrane collaboration tool will be used (Table 8.5.a in the Cochrane Handbook for Systematic Reviews of Interventions). A judgment of the possible risk of bias on each of the six domains will be made from the extracted information, rated as “high risk” or “low risk.” If there is insufficient detail reported in the study, the risk of bias as “unclear” will be judged. We will compute graphic representations of potential bias within and across studies using Revman 5.3 (Review Manager 5.3) [36].

Whenever there is a disagreement, a third reviewer will be used as an arbitrator. The entire process will be undertaken independently by two reviewers.

### Evidence synthesis

After reading the articles, if there is a very high heterogeneity, metanalysis will not be performed. In case of homogeneity, metanalysis will be conducted.

Dichotomous data will be compared with odds ratio (OR) and 95% confidence intervals (CI). Continuous outcomes will be analyzed using weighted mean differences or standardized mean differences when different measurement scales are used. The results will be presented at the Forest graph format. Skewed data and non-quantitative data will be presented descriptively.

Heterogeneity will be assessed using the *I*^2^ statistic. To determine the level of heterogeneity, Cochrane classification for *I*^2^ values will be used [37].

It will be explained the source of heterogeneity by subgroup analysis or sensitivity analysis using the following treatment effect modifiers variables: age, geographic region of study, type of study, study language, instruments used to evaluate quality of life, domains contained in quality of life instruments, type of dance, dance practice period, participants’ pathology, risk of study bias, and method of analysis of the results. In this case, metanalyses will be conducted by using random effects method (DerSimonian and Laird), because confidence intervals for the average intervention effect will be wider and corresponding claims of statistical significance will be more conservative [38].

If studies are sufficiently homogeneous in terms of design and comparator, metanalyses will be conducted by using a fixed-effect method (Mantel-Haenszel). [38–40]. Three different tools will be used to assess meta-biases such as publication bias and outcome reporting bias. If 10 or more studies are available, the potential for publication bias through funnel plots will be explored. Additionally, Begg and Mazumdar’s test and Egger’s test will be used to assess small study effects. Finally, for completing metanalyses, RevMan 5.3 will be used [41].

A systematic narrative synthesis will be provided with information presented in the text and tables to summarize and explain the characteristics and findings of the included studies. The narrative synthesis will explore the relationship and findings both within and between the included studies. Finally, a “summary of findings” table as described in the Cochrane handbook will be prepared. It will be used the Grading of Recommendations Assessment, Development, and Evaluation (GRADE) approach for describing the quality of relevant evidence, if applicable.

### Amendments to protocol

Any substantive amendments to this protocol will be registered with PROSPERO as they occur and documented in the final publication.

### Dissemination

We will publish review results in an international peer-reviewed journal and will report results according to the Preferred Reporting Items for Systematic Reviews and Meta-Analyses (PRISMA) statement. A flowchart will be used to display the selection of articles with reasons for exclusion. Study characteristics and measured outcomes will be compiled into summary tables. An Egger’s plot will be included to examine potential publication bias in the selected studies. If a meta-analysis is possible, results will be presented in a forest plot. The current protocol follows the Preferred Reporting Items for Systematic Review and Meta-Analysis Protocols (PRISMA-P) statement (see Additional file 1).

We will also disseminate results to the research community and relevant key stakeholders through presentations at relevant academic and non-academic meetings and via social media. If findings are found to be interesting to the wider public, we will disseminate them via mass media.

## Discussion

As far as we know, this is the first systematic review related to dance practice and quality of life of adult women. We hope that this study contributes to the discussion of the importance of dance as an option to improve quality of life and also to the discussion about women in society. In addition, the research will be part of a doctoral thesis, articles, posters, and discussions which may instigate further discussions on the subject in the academic community and, consequently, the preparation of proposals designed to enhance quality of life and health of women in general.

There are studies that relate dance, health, and well-being. Many of them feature very positive results from the use of dance in treatment of several diseases and also in the process of people socialization. Improvements in health and well-being directly or indirectly affect quality of life. Therefore, studies focused on the relationship between dance and quality of life are pertinent and necessary, especially about the feminine universe. It is a public that has grown much demographically, influences globally all the areas of the labor market, and needs special attention in terms of levels of stress and number of severe and chronic diseases developed [42–45].

This systematic review may present some potential limitations. One of them may be the fact that we will not include studies that were presented at scientific events and have not been published in scientific journals yet. Also, studies in languages other than English, Portuguese, and Spanish will not be included. Another limitation may be the presence of an investigation bias, given that one of the researchers is a dance practitioner and can unconsciously influence the exposure and discussion of the results inside the review.

## Additional files

Additional file 1: Preferred Reporting Items for Systematic Reviews and Meta-Analysis Protocols (PRISMA-P) 2015 checklist. (DOC 96 kb)

Additional file 2: Search strategies MEDLINE. MEDLINE search strategy. (DOCX 11 kb)

## Abbreviations

BVS: Biblioteca Virtual em Saúde
CAPES: Coordenação de Aperfeiçoamento de Pessoal de Nível Superior
DeCS: Descritores em Ciências da Saúde
Emtree: Embase Thesaurus
FEBRASGO: Federação Brasileira das Associações de Ginecologia e Obstetrícia
GRADE: Grading of Recommendations Assessment, Development, and Evaluation
MeSH: Medical Subject Headings
PRISMA: Preferred Reporting Items for Systematic Reviews and Meta-Analyses
PRISMA-P: Preferred Reporting Items for Systematic Review and Meta-Analysis Protocols
SciELO: Scientific Electronic Library Online
SF-36: Medical Outcomes Study 36-Item Short-Form Health Survey
WHO: World Organization of Health
WHOQOL: World Health Organization Quality of Life Measures
