# Supplementary material for: Quality of life of women who practice dance: a systematic review protocol
Source: Syst Rev. 2018 Jul 10;7:92. doi: 10.1186/s13643-018-0750-5 (PMC6040078; doi:10.1186/s13643-018-0750-5)
Supplement: Supplementary file 2 — Search strategies MEDLINE. MEDLINE search strategy. (DOCX 11 kb) [file 13643_2018_750_MOESM2_ESM.docx]

**Additional file 2 : Example of search stratgey in MedLine**

*(("quality of life"[MeSH Terms] OR ("quality"[All Fields] AND "life"[All Fields]) OR "quality of life"[All Fields]) AND ("dancing"[MeSH Terms] OR "dancing"[All Fields] OR "dance"[All Fields])) AND "Quality of life"[All Fields] AND "women"[All Fields] AND "dance"[All Fields]*
